# Changes in Plasma Copeptin Levels during Hemodialysis: Are the Physiological Stimuli Active in Hemodialysis Patients?

**DOI:** 10.1371/journal.pone.0127116

**Published:** 2015-05-14

**Authors:** Esmée M. Ettema, Johanna Kuipers, Solmaz Assa, Stephan J. L. Bakker, Henk Groen, Ralf Westerhuis, Carlo A. J. M. Gaillard, Ron T. Gansevoort, Casper F. M. Franssen

**Affiliations:** 1 Department of Internal Medicine, Division of Nephrology, University Medical Center Groningen, University of Groningen, Groningen, The Netherlands; 2 Dialysis Center Groningen, Groningen, The Netherlands; 3 Department of Epidemiology, University Medical Center Groningen, University of Groningen, Groningen, The Netherlands; Medical University of Graz, AUSTRIA

## Abstract

**Objectives:**

Plasma levels of copeptin, a surrogate marker for the vasoconstrictor hormone arginine vasopressin (AVP), are increased in hemodialysis patients. Presently, it is unknown what drives copeptin levels in hemodialysis patients. We investigated whether the established physiological stimuli for copeptin release, i.e. plasma osmolality, blood volume and mean arterial pressure (MAP), are operational in hemodialysis patients.

**Methods:**

One hundred and eight prevalent, stable hemodialysis patients on a thrice-weekly dialysis schedule were studied during hemodialysis with constant ultrafiltration rate and dialysate conductivity in this observational study. Plasma levels of copeptin, sodium, MAP, and blood volume were measured before, during and after hemodialysis. Multivariate analysis was used to determine the association between copeptin (dependent variable) and the physiological stimuli plasma sodium, MAP, excess weight as well as NT-pro-BNP immediately prior to dialysis and between copeptin and changes of plasma sodium, MAP and blood volume with correction for age, sex and diabetes during dialysis treatment.

**Results:**

Patients were 63±15.6 years old and 65% were male. Median dialysis vintage was 1.6 years (IQR 0.7–4.0). Twenty-three percent of the patients had diabetes and 82% had hypertension. Median predialysis copeptin levels were 141.5 pmol/L (IQR 91.0–244.8 pmol/L). Neither predialysis plasma sodium levels, nor NT-proBNP levels, nor MAP were associated with predialysis copeptin levels. During hemodialysis, copeptin levels rose significantly (p<0.01) to 163.0 pmol/L (96.0–296.0 pmol/L). Decreases in blood volume and MAP were associated with increases in copeptin levels during dialysis, whereas there was no significant association between the change in plasma sodium levels and the change in copeptin levels.

**Conclusions:**

Plasma copeptin levels are elevated predialysis and increase further during hemodialysis. Volume stimuli, i.e. decreases in MAP and blood volume, rather than osmotic stimuli, are associated with change in copeptin levels during hemodialysis.

## Introduction

Arginine vasopressin (AVP) is as vasoconstrictor hormone involved in blood pressure regulation [1,2]. In hemodialysis patients, interdialytic plasma AVP levels are higher compared with healthy individuals [3–6]. However, during hemodialysis, little or no increase in AVP levels has been reported despite decreases in blood pressure and blood volume during most hemodialysis sessions [7]. In the literature it has been speculated that deficient or inadequate release of AVP could play a role in the pathogenesis of intradialytic hypotension [2,6,8].

Routine measurement of AVP is hampered by preanalytical variability and instability in plasma, leading to short ex-vivo half-life [9,10]. Copeptin, the C-terminal part of the AVP precursor, is a more stable analyte and considered to be a good surrogate marker of AVP [11–13]. Like AVP, plasma levels of copeptin are increased in patients with chronic kidney disease and in patients on hemodialysis compared with healthy individuals [14–16], in whom copeptin ranges between 1 and 12 pmol/L [10]. Interestingly, higher copeptin concentration has been identified as a strong risk marker for cardiovascular disease and mortality in hemodialysis patients [14–16].

Currently, it is unknown why hemodialysis patients have elevated AVP and copeptin levels. It has been suggested that elevated predialysis plasma copeptin levels originate from the hemodialysis treatment itself as a consequence of intradialytic volume depletion [14]. However, the course of plasma copeptin levels during hemodialysis has not been studied before. Not only hypovolemia, but also changes in blood pressure and plasma osmolality during hemodialysis may influence the intradialytic course of copeptin levels [10]. The goal of this study was to explore whether the physiological stimuli for copeptin release as a surrogate marker of AVP, i.e. changes in osmolality, blood volume and blood pressure, are operational in hemodialysis patients. We hypothesized that intradialytic decreases in blood volume and blood pressure and an increase in plasma sodium concentration are associated with increases in copeptin. Knowing and understanding the course of copeptin levels during hemodialysis may help to elucidate the mechanisms that lead to increased plasma copeptin levels in hemodialysis patients and understand the role of AVP during hemodialysis with respect to hemodynamic stability.

## Methods

### Patients

Included were patients from the University Medical Center Groningen and the Dialysis Center Groningen who participated in an observational study on the prevalence and determinants of hemodialysis-induced regional left ventricular systolic dysfunction [17]. Patients were eligible for study inclusion when they were 18 years or older, were on a thrice-weekly 4 hour hemodialysis schedule and had been treated with hemodialysis for more than three months.

The study was performed in accordance with the principles of the Declaration of Helsinki and was approved by the Medical Ethics Committee (METc) of the University Medical Center Groningen. All patients gave written informed consent to the study, which was performed between March of 2009 and March of 2010.

### Study protocol

Patients were studied at the first hemodialysis treatment of the week after the longest interdialytic interval. Blood pressure and heart rate were measured using an automatic oscillometric monitor that was incorporated in the hemodialysis apparatus at 30 minutes predialysis, every 30 minutes during hemodialysis until 210 minutes on dialysis and post-dialysis after termination of the hemodialysis session, including the return of the blood and removal of the dialysis needles.

Mean arterial blood pressure (MAP) was calculated as ((2xdiastolic blood pressure) + systolic blood pressure)/3.

Blood samples for the analysis of plasma levels of copeptin, sodium and NT-proBNP were collected from the arterial line at the initiation of hemodialysis, after 60 and 180 minutes on hemodialysis and at the end of the hemodialysis treatment just before the return of the blood from the extracorporeal circuit to the patient to exclude dilution by saline. Plasma samples were stored at -80°C until analysis.

Excess weight at the start of hemodialysis was calculated as predialysis weight minus dry weight of the individual patient. Prescriptions regarding dry weight were made by the nephrologists during their weekly visit to the participating patients. Dry weight was evaluated clinically (peripheral edema, signs of pulmonary congestion, intradialytic and interdialytic blood pressure course, muscle cramps) in combination with the cardio-thoracic ratio on chest radiography, which is performed twice yearly as standard of care.

### Hemodialysis treatment

All patients were on bicarbonate dialysis with a low-flux polysulfone hollow-fiber dialyser F8 (Fresenius Medical Care, Bad Hamburg, Germany). Blood flow and dialysate flow rates were 250–350 ml/min and 500 ml/min, respectively. Dialysate temperature was 36.0°C. Dialysate composition was sodium 139 mmol/L, potassium 1.0 or 2.0 mmol/L, calcium 1.5 mmol/L, magnesium 0.5 mmol/L, chloride 108 mmol/L, bicarbonate 34 mmol/L, acetate 3.0 mmol/L, and glucose 1.0 g/dl.

Dialysate conductivity and ultrafiltration rate were kept constant during the dialysis session.

Patients received a light meal at 60 minutes on hemodialysis. Patients were dialyzed in supine position which excluded an effect of posture changes on blood volume.

### Laboratory procedures and definitions

Blood samples for the determination of copeptin were collected in ethylenediaminetetraacetic acid (EDTA) tubes and copeptin was measured by a sandwich immunoassay (CT-proAVP LIA; ThermoFisher Scientific, B.R.A.H.M.S. Biomarkers, Henningsdorf/Berlin, Germany) as described previously [11,18]. Blood samples for the determination of sodium were collected in heparin-coated tubes and measured with the indirect method of ion-selective electrode on a Roche Modular (Hitachi, Tokyo, Japan). NT-proBNP was measured by the electrochemiluminescence immunoassay (Roche Diagnostics, Mannheim, Germany). The change in blood volume was calculated from the change in hematocrit ([(Ht_0_/Ht_1_)-1]x100) with Ht_0_ representing the hematocrit at the start of hemodialysis and Ht_1_ representing the hematocrit during hemodialysis. Ultrafiltration rate was calculated by dividing ultrafiltration volume by dialysis session length and target weight.

Diabetes mellitus was defined as fasting blood glucose >6 mmol/l or the use of antidiabetic drugs. Hypertension was defined as a predialysis systolic blood pressure >140 mmHg and/or a diastolic blood pressure >90 mmHg or the use of antihypertensive drugs. Cardiovascular history was defined as any history of ischemic heart disease, congestive heart failure, coronary artery bypass grafting, percutaneous coronary intervention, stroke or peripheral vascular disease. The presence of residual renal function was indicated by urinary volume ≥200 ml/day.

### Statistical analysis

Normally distributed variables are reported as mean ± SD. Variables with a skewed distribution are presented as median and interquartile range (IQR). Categorical data is reported as number and percentage. Continuous variables were analyzed with One Way ANOVA (post-hoc test with Bonferroni correction), paired t-test, Wilcoxon Signed Rank test and Wilcoxon Ranked Sum (Mann-Whitney U) test when appropriate. Categorical variables were analyzed with the Kruskal-Wallis test. Non-normally distributed variables were transformed to achieve normality.

Baseline characteristics are given for the overall population and according to tertiles of copeptin concentration. Plasma copeptin tertiles were stratified for gender since gender is known to affect plasma copeptin levels [11]. Correlations were calculated using Spearmans’ rho for skewed data.

Multiple regression analysis was used to analyze the association between the putative stimuli of copeptin release and (changes in) plasma copeptin levels. In these analyses, postdialysis values of copeptin, MAP and sodium were corrected for the baseline value. Effects of these variables were corrected for factors selected from the univariate comparison, based on a p-value <0.157 using backward stepwise selection as advised in literature [19]. To test the robustness of our findings various sensitivity analyses were performed. First, we investigated the intradialytic course of copeptin with a correction for hemoconcentration. Second, we tested whether changes in copeptin levels differed between diabetic and non-diabetic patients and between patients with and without residual diuresis by inserting an interaction term for diabetes and residual diuresis, respectively, and the stimuli of copeptin in the multivariable models. Analyses were performed with SPSS version 20.0 and GraphPad Prism version 5.0. P-values of <0.05 (two-tailed) were considered statistically significant.

## Results

### Patients

Characteristics of participating patients are shown in Table 1. The mean (±SD) age was 63 ± 15.6 years and 65% were male (Table 1). The median dialysis vintage was 1.6 years (IQR 0.7–4.0) and 41% of the patients had residual diuresis. Twenty-three percent of the patients had diabetes mellitus and 82% of the patients had hypertension. Causes of renal failure were hypertension (n = 18), diabetes mellitus (n = 14), autosomal dominant polycystic kidney disease (n = 14), glomerulonephritis (n = 13), focal segmental glomerulosclerosis (n = 10), chronic obstructive nephropathy (n = 6), IgA nephropathy (n = 4), chronic pyelonephritis (n = 3) and miscellaneous causes (n = 17) or unknown etiology (n = 9).

**Table 1:** Patient characteristics according to sex-stratified tertiles of predialysis copeptin levels.

	All patients		1st tertile; M: ≤127.7 pmol/L; F: ≤59.5 pmol/L		2nd tertile; M: >127.7 to ≤233.3 pmol/L; F: >59.5 to ≤161.0 pmol/L		3rd tertile; M: >233.3 pmol/L; F: >161.0 pmol/L		*P-value between tertiles
		N		N		N		N	
Age, years	63 ± 15.6	108	69 ± 14.9	35	59 ± 15	37	60 ± 15	36	0.01**
Male	70 (65)	108	23/12 (65.7/34.3)	35	24/13 (64.9/35.1)	37	23/13 (63.9/36.1)	36	0.9
Dialysis vintage, years	1.6 [0.7–4.0]	108	1.8 [0.7–4.1]	35	1.2 [0.4–3.5]	37	2.0 [0.9–4.2]	36	0.11
Residual diuresis	39 (40.6)	96	14 (46.7)	30	17 (50.0)	34	8 (25.0)	32	0.09
BMI, kg/m^2^	25.9 ± 4.4	108	25.4 ± 4.1	35	26.6 ± 4.9	37	25.6 ± 4.1	36	0.45
Diabetes mellitus	25 (23.1)	108	4 (11.4)	35	10 (27.0)	37	11 (30.6)	36	0.13
HbA1c, %	5.4 [5.0–6.0]	89	5.4 [5.1–5.7]	28	5.4 [5.2–5.9]	34	5.6 [4.9–6.4]	27	0.63
Hypertension	89 (82.4)	108	30 (85.7)	35	32 (86.5)	37	27 (75.0)	36	0.36
Cardiovascular history	25 (23.1)	108	10 (28.6)	35	5 (13.5)	37	10 (27.8)	36	0.23
Ultrafiltration volume, mL	2549 ± 779	108	2396 ± 796	35	2679 ± 641	37	2565 ± 882	36	0.31
Ultrafiltration rate (mL/kg/h)	8.5 ± 2.6	108	8.5 ± 2.9	35	8.4 ± 2.1	37	8.7 ± 2.8	36	0.82
Medication									
ACEi	10 (9.4)	108	2 (1.9)	34	3 (2.8)	36	5 (4.7)	36	0.50
ARB	14 (13)		7 (6.8)	35	3 (2.5)	37	4 (3.7)	36	0.30
Beta blocker	62 (57.4)		21 (19.4)	35	19 (17.6	37	22 (20.4)	36	0.66
CCB	14 (13.2)		7 (6.6)	34	5 (4.7)	36	2 (1.9)	36	0.18
Diuretics	8 (7.5)		3 (2.8)	34	5 (4.7)	36	0 (0)	36	0.08
Statins	20 (18.9)		7 (6.5)	34	5 (4.7)	36	8 (7.5)	36	0.64
Aspirin	58 (54.7)		22 (20.8)	34	18 (17.0)	36	18 (17.0)	36	0.37
Excess weight, k**g**	2.2 [1.3–3.3]	108	1.9 [1.0–2.9]	35	2.4 [1.9–3.6]	37	2.4 [1.2–3.3]	36	0.10
SBP, mmHg	140 ± 25	107	144 ± 24	35	143 ± 22	36	134 ± 28	36	0.15
DBP, mmHg	80 ± 16	107	78 ± 15	35	83 ± 15	36	79 ± 19	36	0.49
MAP, mmHg	100 ± 18	107	100 ± 15	35	103 ± 16	36	97 ± 21	36	0.41
Plasma sodium, mmol/l	138 ± 3	106	138 ± 3	35	138 ± 4	37	139 ± 2	34	0.44
NT-proBNP, pg/ml	3906 [1708–8388]	108	4029 [1594–10187]	35	2889 [1136–6425]	37	4457 [2689–9918]	36	0.14

Continuous variables are represented as mean ± SD, skewed variables as median and interquartile range (IQR) and categorical variables as number and percentage within tertile. The number of the included patients is indicated.

* P-value indicates differences between tertiles.

** Post-hoc test of age showed a significant difference between tertile 1 and 2 (p = 0.02) and between tertile 1 and 3 (p = 0.03).

**Abbreviations:** BMI: body mass index; DBP: diastolic blood pressure; F: females; M: males; MAP: mean arterial blood pressure; NT-proBNP: N-terminal pro-brain natriuretic peptide; SBP: systolic blood pressure.

### Predialysis copeptin levels and physiological stimuli

The median predialysis plasma copeptin level was 141.5 pmol/L (IQR 91.0–244.8). Males had significantly higher plasma copeptin levels than females (161.5 (IQR 104.8–254.3) pmol/L versus 107.0 (IQR 34.9–182.8) pmol/L, respectively; p = 0.02). Patients in the first (lowest) tertile of copeptin levels were significantly older than the patients in the second and third (highest) tertile (Table 1) and there was a significant correlation between predialysis copeptin levels and age (Spearman’s r = -0.261; p = 0.01).

When adjusted for age, sex and diabetes, there was no association of predialysis copeptin levels with predialysis plasma sodium concentration, MAP, excess weight at the start of hemodialysis and NT-proBNP levels. If associations of plasma sodium concentration, MAP, excess weight at the start of hemodialysis and NT-proBNP levels were also adjusted for each other (fully adjusted model), a trend was seen for an association of predialysis plasma copeptin levels with predialysis MAP and levels of NT-proBNP (Table 2).

**Table 2:** Predialysis association between putative determinants and plasma copeptin levels.

Determinants	Multivariate linear regression							
	Model 1				Model 2			
	B	Beta_standardized_	CI	P-value	B	Beta_standardized_	CI	P-value
Predialysis sodium (mmol/l)	0.134	0.098	-0.121; 0.390	0.30	0.183	0.131	-0.081; 0.448	0.17
Predialysis MAP (mmHg)	-0.022	-0.086	-0.071; 0.027	0.37	-0.045	-0.165	-0.098; 0.008	0.09
Excess weight at start of dialysis (kg)	0.062	0.006	-1.989; 2.113	0.9	0.464	0.044	-1.691; 2.618	0.67
Predialysis NT-proBNP (ng/l)	0.525	0.152	-0.120; 1.170	0.11	0.612	0.177	-0.049; 1.273	0.07

Model 1: adjustment for age, sex and diabetes.

Model 2: as model 1 and additionally adjusted for the other determinants included in this model.

**Abbreviations:** CI: confidence interval of B; MAP: mean arterial pressure; NT-proBNP: N-terminal pro-B type natriuretic peptide.

### Intradialytic change of plasma copeptin levels and physiological stimuli

**Fig 1:**
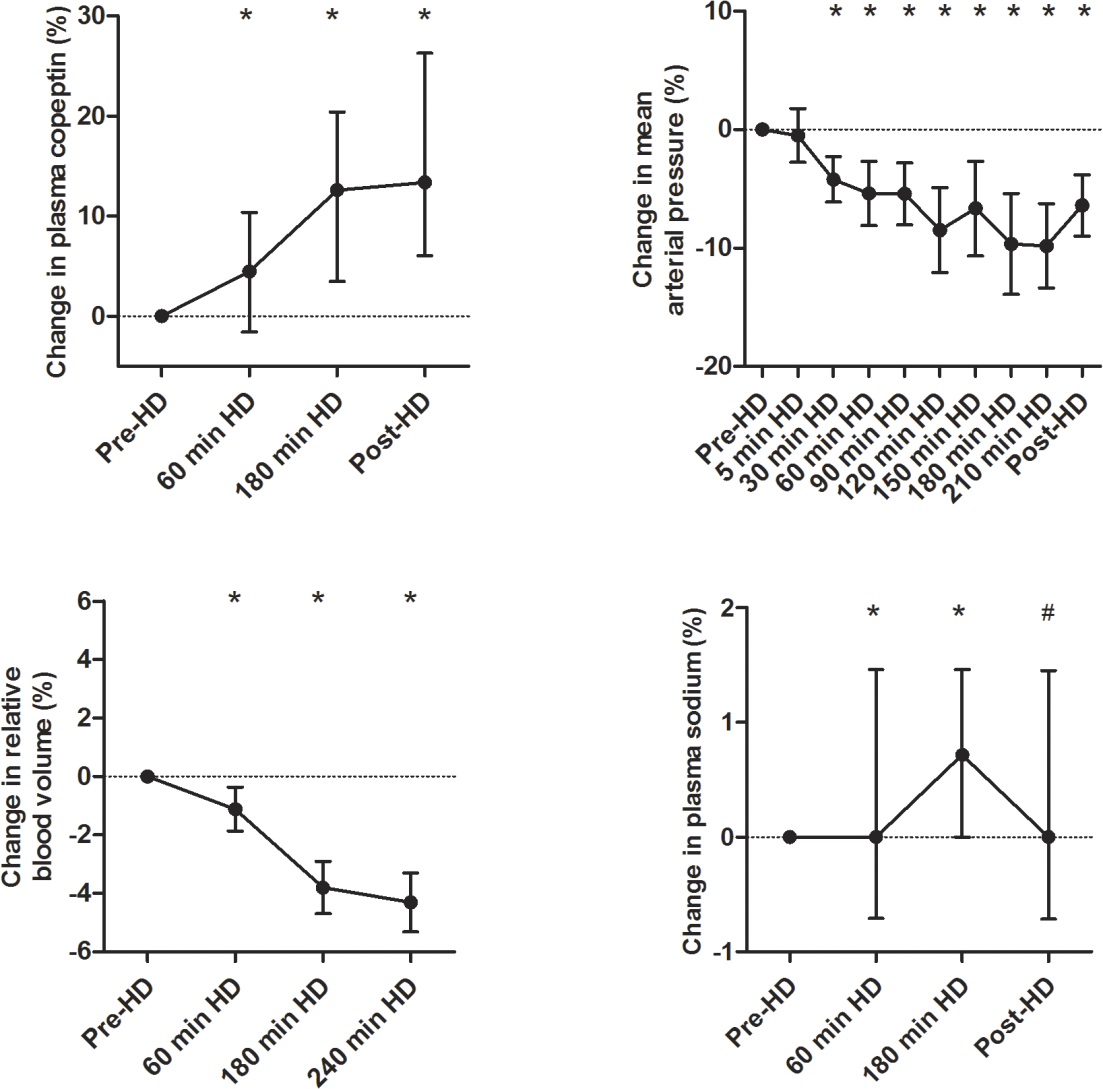
Intradialytic course of the change in copeptin, mean arterial pressure, blood volume and plasma sodium levels (%). Depicted are median and interquartile range (IQR) for copeptin and sodium and mean and 95% confidence intervals for MAP and blood volume. #Denotes p<0.05 compared with the predialysis value. *Denotes p<0.01 compared with the predialysis value.

The courses of the intradialytic change in copeptin, MAP, blood volume, and plasma sodium are shown in Fig 1. During hemodialysis, systolic blood pressure decreased from 140±25 mmHg predialysis to 126±25 mmHg at 210 minutes on dialysis and to 132±26 mmHg postdialysis. Diastolic blood pressure decreased from 80±16 mmHg predialysis to 71±16 mmHg at 210 minutes on dialysis and to 73±14 mmHg postdialysis and MAP decreased from 101±18 mmHg predialysis to 90±17 mmHg at 210 minutes on dialysis and to 93±16 mmHg postdialysis (all p<0.01). There was only a modest, but significant, increase in heart rate during hemodialysis (from 74±14 to 77±14 bpm at 210 minutes on dialysis and to 80±16 bpm postdialysis, both p<0.01). Relative blood volume decreased significantly during hemodialysis (to -4.4±5.3%; p<0.01). Plasma sodium levels increased slightly but significantly during hemodialysis (from 138.2±3.3 mmol/L predialysis to 138.8±1.9 mmol/L postdialysis; p<0.05).

Plasma copeptin levels increased significantly during hemodialysis: from 141.5 pmol/L (IQR 91.0–244.8) to 148.0 pmol/L (93.0–254.0) at 60 minutes intradialysis, to 155.0 pmol/L (93.9–279.0) at 180 minutes intradialysis and to 163.0 pmol/L (96.0–296.0) at the end of dialysis (p<0.01 for the overall change).

Multivariate analysis showed a significant association of the intradialytic change in copeptin and the change in MAP and blood volume when adjusted for age, sex, diabetes and predialysis copeptin levels. These associations remained significant when we additionally adjusted for plasma sodium concentration and MAP (Table 3). In contrast, change in plasma sodium was not associated with change in copeptin during hemodialysis.

**Table 3:** Association between the intradialytic change in the putative stimuli and the change in plasma copeptin levels during hemodialysis.

Determinants	Multivariate linear regression							
	Model 1				Model 2			
	B	Beta_standardized_	CI	P-value	B	Beta_standardized_	CI	P-value
Δ sodium (mmol/l)	-0.028	-0.010	-0.157; 0.100	0.66	-0.003	-0.001	-0.157; 0.150	0.9
Δ MAP (mmHg)	-0.025	-0.083	-0.045; -0.005	0.02	-0.015	-0.051	-0.030; 0.000	0.04
Δ RBV (%)	-0.049	-0.050	-0.085; -0.014	0.01	-0.057	-0.057	-0.098; -0.016	0.01

Model 1: adjustment for age, sex, diabetes and predialysis copeptin levels.

Model 2: as model 1 and additionally adjusted for the other determinants included in this model.

Δ: from predialysis to postdialysis for sodium and RBV, for predialysis to 210 minutes intra-dialysis for MAP, with correction for predialysis sodium and predialysis MAP.

**Abbreviations:** CI: confidence interval of B; MAP: mean arterial pressure; RBV: relative blood volume.

### Sensitivity analyses

To exclude a possible effect of ultrafiltration-induced intravascular volume change on the intradialytic change in plasma copeptin levels, we corrected the intradialytic course of plasma copeptin levels for hemoconcentration according to Schneditz *et al* [20]. Despite this, the increase in plasma copeptin levels during hemodialysis remained significant (p<0.01). When the change in relative blood volume was calculated based on the change in albumin as an alternative marker of hemoconcentration, results of the multivariate analysis remained similar (data not shown).

The results of the multivariate analyses remained similar for the intradialytic change in MAP (β_standardized_ = -0.051, p = 0.04) and plasma sodium (β_standardized_ = -0.007, p = 0.80), but, as to be expected, the change in blood volume was no longer significantly associated with change in copeptin levels (β_standardized_ = 0.021, p = 0.31).

Although predialysis plasma copeptin levels were higher in diabetic patients in comparison with non-diabetic patients (188.0 pmol/L (IQR 119.5–284.0) versus 131.0 pmol/L (IQR 85.0–242.0), respectively (p = 0.06), plasma copeptin levels increased significantly and to the same extent in both diabetic (+12.8%) and non-diabetic (+13.0%) patients to 212.0 pmol/L (IQR 125.5–319.5) and 148.0 pmol/L (IQR 82.7–283.3), respectively. To assess whether diabetic and non-diabetic patients respond in a similar fashion to the physiological volume, pressure and osmotic stimuli, we also tested whether there was an interaction between the determinants of copeptin during hemodialysis and diabetes. No statistically significant interactions for diabetes status with plasma sodium concentration, MAP or blood volume were found.

Thirty-nine patients had residual diuresis. Plasma copeptin levels tended to be higher in patients without residual diuresis compared with patients with residual diuresis (167.0 pmol/L (IQR 95.3–261.0) versus 134.0 pmol/L (IQR 85.2–187.0); p = 0.09) and predialysis copeptin levels correlated significantly with residual diuresis; the higher daily residual urinary production, the lower predialysis plasma copeptin levels (Spearman’s r = -0.246; p = 0.02). On the other hand, there was no correlation between predialysis copeptin levels and dialysis vintage (Spearman’s r = 0.071; p = 0.47). To assess whether patients with and without residual diuresis respond similarly to the physiological volume, pressure and osmotic stimuli, we also tested whether there was an interaction between the determinants of copeptin during hemodialysis and residual diuresis. No statistically significant interactions were found between for residual diuresis and plasma sodium concentration, MAP and blood volume. Results were similar when residual renal clearance instead of residual diuresis was used in the analyses (data not shown).

## Discussion

The present study shows that plasma copeptin levels are elevated predialysis and rise during hemodialysis. Volume stimuli, i.e. decreases in blood pressure and blood volume, rather than the change in plasma sodium level as an osmotic stimulus, were significantly associated with the intradialytic change in plasma copeptin levels.

In this study, predialysis plasma copeptin levels were markedly elevated in comparison with healthy persons [11,21]. This is in agreement with the findings of other studies in hemodialysis patients [14,16]. In our cohort, predialysis plasma copeptin levels were even higher than in these previous studies [14,16]. This could be explained by differences in patient characteristics, in particular a longer dialysis vintage in our study as compared with previous studies in hemodialysis patients [14,16]. In these studies, a significant association between higher predialysis plasma copeptin levels and a longer dialysis vintage was found. If the in-vivo half life of copeptin is long enough, the dialysis procedure itself could lead to accumulation since copeptin increased during hemodialysis, as is shown in this study, resulting in gradually increasing copeptin levels in the long-term. This could especially be the case with low-flux hemodialysis, as was used in the present study since one would expect less removal of copeptin with low-flux hemodialysis in comparison with high-flux hemodialysis [16]. In addition, it has been suggested that the high predialysis copeptin levels are attributable to accumulation due to impaired renal clearance [14,16]. Our finding of an inverse correlation between predialysis copeptin and residual diuresis and residual clearance supports this assumption. In non-dialysis patients with chronic kidney disease [15] and healthy men [21] also an inverse correlation between eGFR and copeptin was found. It is not clear if AVP is also affected by renal function and to what extent in comparison with copeptin. The higher proportion of males in our study as compared with the other two studies probably also contributed to the higher copeptin levels. It has previously been shown in healthy individuals that males have higher copeptin levels than females [11,21]. To our knowledge, our study is the first to show that this is also the case in hemodialysis patients.

We observed a trend for the association between predialysis copeptin levels and NT-proBNP levels and MAP. In previous studies, a significant association between copeptin and NT-proBNP was found [14,16]. In contrast, the finding of a trend for the association between a lower MAP and higher copeptin concentrations is not in line with previous studies, in which a weak but significant correlation was observed between higher systolic blood pressure and higher copeptin levels by Fenske *et al* [14], while Artunc *et al* did not find any association between predialysis copeptin levels and blood pressure [16]. These divergent associations could be explained by differences in predialysis blood pressure, volume status and the proportion of patients with diabetes mellitus.

The suggestion that the dialysis procedure itself could contribute to the markedly elevated copeptin levels that are observed in hemodialysis patients has been proposed previously by Fenske *et al*. This group suggested that volume depletion during hemodialysis might be responsible for the elevated AVP levels in hemodialysis patients [14]. Notably, such a hemodialysis-associated increase in copeptin levels can only contribute to elevated predialysis copeptin levels if the half-life of copeptin is relatively long. Unfortunately, data on the in-vivo half-life of copeptin are lacking in hemodialysis patients. If future studies would confirm that the in-vivo half life of copeptin is long enough, allowing the hemodialysis procedure to significantly contribute to elevated predialysis copeptin levels in hemodialysis patients, this might explain, at least in part, the association between elevated plasma copeptin levels and adverse outcome. Large reductions in blood pressure during hemodialysis [22,23] and higher ultrafiltration rates [24], probably translating in more pronounced hypovolemia, are associated with a greater risk of all-cause mortality and cardiovascular events. As this study shows, intradialytic reductions in blood pressure and blood volume are also associated with a rise in copeptin levels and copeptin, in turn, is associated with an increased risk for cardiovascular events and all-cause mortality in hemodialysis patients [14]. It remains to be studied whether copeptin is merely a marker of hemodynamic stress induced by hemodialysis, or contributes to the increased risk for adverse events in hemodialysis patients.

This is the first study that measured copeptin levels not only before but also during and at the end of hemodialysis. We noted that plasma copeptin levels rose significantly during hemodialysis and that the increase remained significant with correction for hemoconcentration as a result of ultrafiltration. The significant association between decreases in blood pressure and blood volume and a rise in copeptin levels indicates that the physiological pressure and volume stimuli for the release of copeptin and thus of AVP are active in hemodialysis patients. Thus, it is unlikely that deficient or inadequate AVP release during hemodialysis is explained by a lack of stimuli during hemodialysis or by insensitivity of the hypothalamus-pituitary-axis, at least not in this cohort. Since copeptin behaves similarly to AVP in the circulation [10], one would expect AVP also to increase during hemodialysis. However, the intradialytic increase of plasma AVP as well as plasma copeptin could well be underestimated as copeptin and AVP are probably removed by dialysis [25] considering its molecular weights of about 5 kDa (11,15] and 1 kDa [26], respectively. Presently, there are no data on the dialyzability of copeptin nor AVP and future studies should investigate whether copeptin and AVP are indeed removed by hemodialysis and if so to what extent. Whether the intradialytic courses of copeptin and AVP are similar also remains to be studied.

In this study, plasma sodium levels increased slightly but significantly during hemodialysis with a dialysate sodium concentration of 139 mmol/l. Remarkably, we found no significant association between the change in copeptin levels and changes in plasma sodium during hemodialysis. Under normal physiological conditions, the secretion of AVP, and thus copeptin, is mainly regulated by changes in plasma osmolality [27–30]. However, in hypotensive states, plasma AVP release is thought to be mainly controlled by the baroreceptorreflex [27] and, in such situations, changes in blood volume may play a more important role in AVP release [28]. On the other hand, we should be cautious with conclusions on the absence of a significant association between the change in plasma sodium concentration and copeptin levels. First, the overall change of plasma sodium concentration was modest. Second, since we did not measure plasma glucose levels, we could not calculate the effective plasma osmolality and, thus, could not relate changes in plasma osmolality with changes in copeptin levels. However, when we analyzed diabetic and non-diabetic patients separately, we found that plasma copeptin levels rose significantly and to the same extent in both groups. Furthermore, there was no statistically significant interaction between diabetes status and plasma sodium concentration, MAP and blood volume in their associations with the change in copeptin concentration. A drawback of the present study is that an objective marker of volume status, like bioimpedance analysis, is lacking. It is intriguing that higher copeptin levels prior to dialysis tended to be associated with higher plasma levels of the volume marker NT-proBNP, whereas during hemodialysis a decrease in relative blood volume was associated with a significant increase in plasma copeptin levels. The former was also found previously: the more volume overloaded patients were, the higher plasma copeptin levels were [14]. We have no explanation for this apparent paradox. Copeptin might also be a volume marker in steady state situations, i.e. before hemodialysis and in the interdialytic interval. More studies on the course of copeptin in the intra- and interdialytic interval are needed.

## Conclusion

Plasma copeptin levels are increased predialysis and show a significant increase during hemodialysis. Intradialytic decreases in blood pressure and blood volume were associated with an increase in plasma copeptin. Thus, volumetric stimuli for copeptin release, i.e. decreases in blood pressure and blood volume are active in hemodialysis patients.

## Supporting Information

S1 Dataset PONE-D-14-58216. (XLSX)
